# Piwi-interacting RNA 775 (piR-775) predicts favorable prognosis and regulates cell cycle and DNA damage response pathways in breast cancer

**DOI:** 10.1186/s40364-025-00856-1

**Published:** 2025-11-04

**Authors:** Bushra Yasin Abohalawa, Hend Ghassan Eldous, Ramesh Elango, Radhakrishnan Vishnubalaji, Sameera Rashid, Khalid Ouararhni, Ayman Al Haj Zen, Nehad M. Alajez

**Affiliations:** 1 College of Health & Life Sciences, Hamad Bin Khalifa University (HBKU), Qatar Foundation (QF), Doha, Qatar; 2 Translational Oncology Research Center (TORC), Qatar Biomedical Research Institute (QBRI), Hamad Bin Khalifa University (HBKU), Qatar Foundation (QF), PO Box 34110, Doha, Qatar; 3 Department of Laboratory Medicine and Pathology (DLMP), Hamad Medical Corporation (HMC), Doha, Qatar; 4 Royal Liverpool University NHS Foundation Trust, Liverpool, UK; 5 Genomics Core Facility, Hamad Bin Khalifa University, Qatar Foundation, P.O. Box 34110, Doha, Qatar

**Keywords:** Biomarkers, Breast cancer, PiRNAs, PiR-775, Non-coding RNAs, DNA damage response, Synthetic lethality, Combination therapy

## Abstract

Piwi-interacting RNAs (piRNAs) are small non-coding RNAs primarily recognized for their roles in germline development and genome stability. However, their contribution to breast cancer remains poorly understood. In this study, we profiled piRNA expression in 96 primary breast cancer tissues and explored the prognostic and functional significance of piR-775. Higher expression of piR-775 was significantly associated with improved relapse-free survival, suggesting its potential as a favorable prognostic biomarker. Functional assays in triple-negative breast cancer (TNBC) models demonstrated that piR-775 overexpression significantly suppressed colony formation and three-dimensional tumor growth. Using an organ-on-chip platform, piR-775 overexpression also inhibited TNBC cell migration and invasion. Transcriptomic analyses following piR-775 re-expression identified multiple downregulated target genes involved in cell cycle progression and DNA damage repair pathways, including POLE, BARD1, TPX2, BIRC5, and XRCC2, which were further validated by RT-qPCR. Integration with CRISPR screen data highlighted 47 essential piR-775 targets required for TNBC survival. Additionally, DNA damage assays indicated increased genomic instability upon piR-775 expression. Notably, combining piR-775 with the PARP inhibitor Olaparib led to enhanced cytotoxic effects, suggesting a synthetic lethal interaction. Furthermore, transcriptomic analysis of breast cancer revealed consistent downregulation of the PIWI proteins PIWIL2 and PIWIL4, indicating disruption of the piRNA–PIWI pathway in breast cancer. Collectively, these findings establish piR-775 as a tumor-suppressive piRNA with both prognostic and therapeutic relevance in breast cancer, particularly in TNBC. By impairing oncogenic signaling and enhancing the efficacy of PARP inhibitors, piR-775 emerges as a promising biomarker and a candidate for combination therapy.

**Supplementary Information:**

The online version contains supplementary material available at 10.1186/s40364-025-00856-1.

To the Editor,

Breast cancer remains a leading cause of cancer-related death among women worldwide. According to GLOBOCAN 2022, 2.3 million new cases and over 666,000 deaths were reported globally, with rising incidence rates noted in the Middle East and North Africa (MENA) region [1, 2]. Among emerging noncoding RNA classes, PIWI-interacting RNAs (piRNAs) have garnered attention for their roles in genome defense, epigenetic regulation, and cancer biology [3, 4]. However, their prognostic significance and functional relevance in breast cancer remain largely unexplored.

To address this gap, we profiled piRNA expression in 96 primary breast cancer tissues using a sequential RNA-Seq mapping pipeline. Reads that did not align to known miRNAs (miRBase v22) were subsequently mapped to piRNAdb v1.7.6, which includes 27,700 annotated piRNAs. Clustering based on the top 200 most variable piRNAs revealed associations with key clinicopathological features, including PAM50 molecular subtype, tumor grade, and patient age (Fig. 1A–B). Differential expression analysis (FDR < 0.05, fold change > 1.5) uncovered widespread piRNA deregulation, with 81 upregulated and 12 downregulated piRNAs distinguishing Basal from Luminal subtypes. Additionally, distinct piRNA expression patterns were observed across different tumor grades and age groups (Fig. S1A–B, Table S1).

**Fig. 1:**
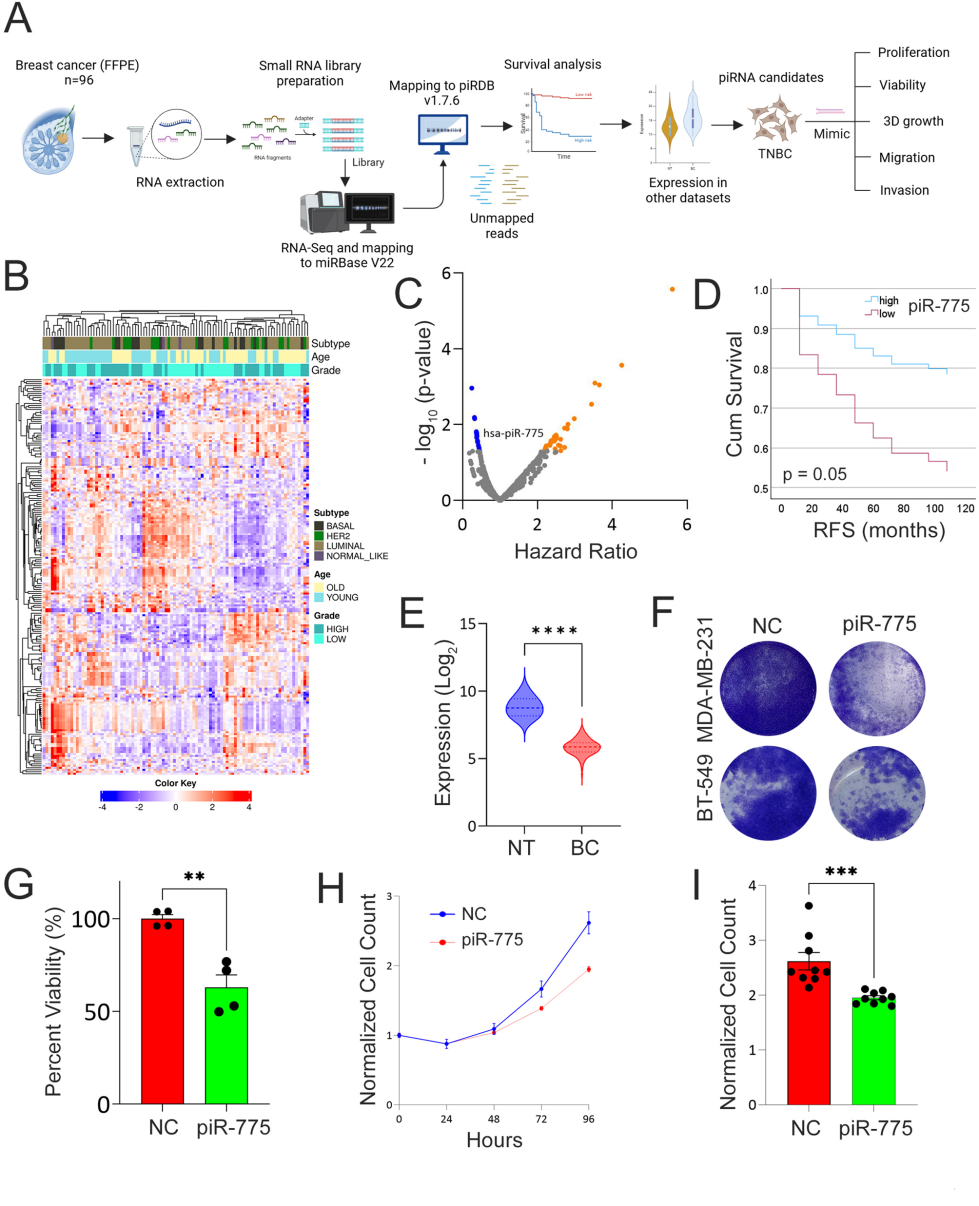
Hierarchical clustering and differential piRNA expression analysis in breast cancer. **(A)** Illustration of overall study design including library preparation, mapping to piRDB, and functional investigations. **(B)** Heatmap depicting clustering of 96 breast cancer patients according to top 200 most variable piRNAs in relation to PAM50 intrinsic subtype classification (Luminal, Basal, HER2, and Normal-like), tumor grade (high vs. low), and age (young vs. old). Color scale depicts the expression level of each piRNA. Each row represents a single piRNA, and each column represents a sample. **(C)** Volcano plot illustrating the set of piRNAs correlating with poor (orange) and better (blue) prognosis in our cohort (*n* = 88). Kaplan Myers was used to calculate hazard ration (HR) and log-rank p value. X-axis indicates HR while y-axis represent -log10(p-value). **(D)** Kaplan Myers survival curve for breast cancer patients stratified into high vs. low based on median piR-775 expression. Log-rank p value was used to calculate significance. **(E)** Validation of downregulated expression of piR-775 in breast cancer (*n* = 103) compared to normal breast (*n* = 11) from PRJNA281709 dataset. **** *p* < 0.00005. **(F)** Representative images illustrating effects of exogenous expression of piR-775 on MDA-MB-231 and BT-549 cell proliferation. **(G)** Quantification of proliferation potential in piR-775 mimic compared to negative control transfected cells in both models. Data are presented as mean ± S.D., *n* = 4. **(H)** Time course cell proliferation of GFP-labelled MDA-MB-231 cells transfected with piR-775 mimic compared to negative control transfected cells. Plates were imaged immediately (T0) and at 24 h intervals over a period of 96 h using Operetta high content imaging system (PerkinElmer). Nine view fields were acquired for each well using a 10x objective. GFP labelled cell numbers were quantified using Harmony^®^ image analysis software. To calculate the cell growth rate, cell count at each time point was normalized to its respective replicate at T0. Data are presented as mean ± S.E.M., *n* = 9. **(I)** Normalized cell count at 96 h in MDA-MB-231 cells transfected with piR-775 mimic compared to negative control transfected cells. Data are presented as mean ± S.E.M., *n* = 9. *** *p* < 0.0005

Relapse-free survival (RFS) analysis in 88 patients with available survival data identified several piRNAs predicting better and worse RFS (Fig. 1C). Among those, piR-775 was significantly associated with better outcome (HR = 0.37, *p* = 0.05), independent of age, grade, or subtype (Fig. 1D) and was also downregulated in tumor vs. normal breast tissues in independent RNA-Seq dataset (Fig. 1E), supporting its tumor-suppressive role and therapeutic potential.

Functional assays confirmed this, where re-expression of piR-775 in TNBC cell lines (MDA-MB-231 and BT-549) significantly reduced colony formation and proliferation (Fig. 1F–G and Fig. S2), with less cytotoxicity in non-tumorigenic MCF10A cells (Fig. S3). High-content imaging revealed impaired proliferation of MDA-MB-231 TNBC cells transfected with piR-775 mimics (Fig. 1H–I), accompanied by hallmarks of apoptosis and necrosis (Fig. S4A–B). Our findings are consistent with other published reports documenting tumor-suppressive effects for other piRNAs, including piR-YBX1, which targets the MAPK-YBX1 axis in TNBC [5], and piR-36712, which impairs colony formation in ER + BC [6].

piR-775 also suppressed migratory and invasive behavior in TNBC. Wound-healing assays showed 64% reduction in migration (Fig. 2A–B), and organ-on-chip analysis demonstrated a 74% reduction in invasion (Fig. 2C–D). Moreover, 3D organotypic growth was markedly suppressed in piR-775–transfected TNBC cells (Fig. S5A–B).

**Fig. 2:**
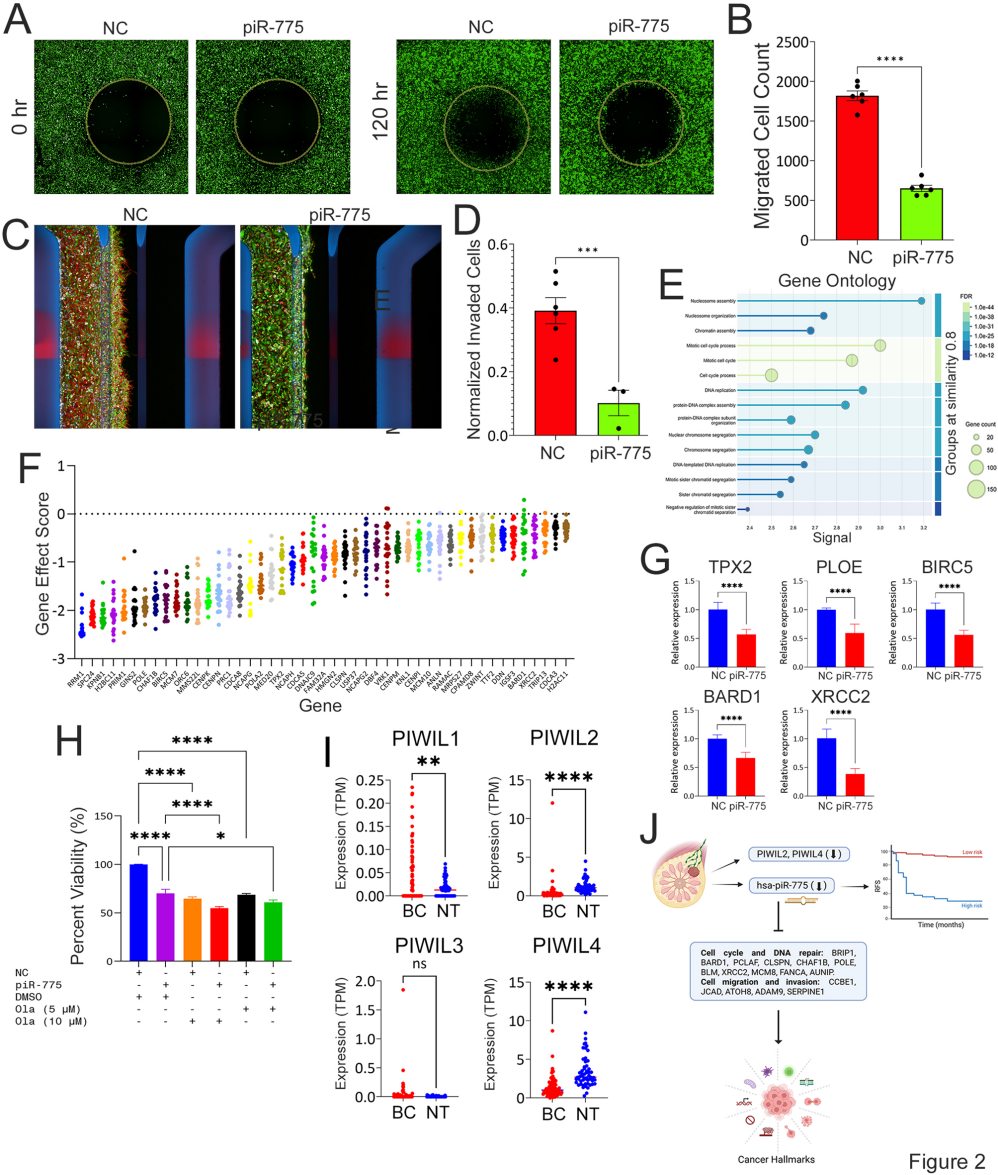
piR-775 suppresses tumor cell migration and invasion and modulates genes involved in cell cycle progression and DNA repair. Representative images showing migration of of EGFP-labeled MDA-MB-231 cells after transfection with non-targeting controls or piR-775 mimics **(A)**. Cells were seeded in 96-well plates with stoppers, cultured overnight, and imaged immediately after stopper removal (T0) and after 120 h using the Operetta High Content Imaging System (PerkinElmer). Images from nine fields per well were stitched for analysis. **B)** Quantification of cell migration at 120 h using Harmony^®^ image analysis software and NIH ImageJ. Data represent mean ± S.E.M. (*n* = 9). ****p < 0.00005. The OrganoPlate^®^-3-lane (Mimetas) microfluidic platform was used to evaluate cancer cell invasion. EGFP-labeled MDA-MB-231 cells were seeded and transfected with NC or piR-775 mimics. Bi-directional flow was maintained throughout the experiment. After 5 days, cells were fixed, stained, and imaged using a high-content imaging system. **C)** Representative images are shown for the NC and piR-775 conditions. At the end of experiment, tumor cell invasion was quantified and normalized to the total seeded cells for each condition **(D)**. Data are presented as mean ± S.E.M. (*n* = 6 for controls, *n* = 3 for piR-775). ***p < 0.0005. **E)** Gene Ontology (GO) enrichment analysis of downregulated genes (fold change ≥ 2.0, *p* < 0.05, FDR), highlighting the top 15 enriched categories. **F)** Analysis of genome-wide CRISPR functional screen data identified 47 piR-775 gene targets as essential in TNBC cell lines (*n* = 22) with gene effect scores < − 0.3. **G)** RT-qPCR validation of selected genes involved in cell cycle and DNA repair pathways (POLE, BARD1, TPX2, BIRC5, XRCC2), showing downregulation upon piR-775 overexpression. Data are presented mean ± S.E.M. (*n* = 6). ****p < 0.00005. **H)** Quantification of colony formation potential of MDA-MB-231 cells transfected with piR-775 as single agent or in combination with the indicated concentrations of Olaparib. Data are presented mean ± S.E.M. (*n* = 8). *p < 0.05; ****p < 0.00005. **I)** Transcript levels of PIWIL1, PIWIL2, PIWIL3, and PIWIL4 in our breast cancer cohort (BC; *n* = 96) compared to normal breast tissue (NT; *n* = 56; from PRJNA251383). Expression values are shown as transcripts per million (TPM), with each dot representing an individual sample. Red dots indicate BC samples, and blue dots indicate NT samples. Statistical analysis was performed using an unpaired two-tailed t-test. **p < 0.01; ****p < 0.0001; ns, not significant. **J)** Schematic overview illustrating the downregulation of PIWIL1, PIWIL4, and piR-775 in modulating TNBC hallmarks through the regulation of key cancer-related genes

To uncover downstream targets, RNA-Seq following piR-775 overexpression identified 639 downregulated and 303 upregulated genes (FC ≥ 2, FDR < 0.05; Fig. S6A, Table S2). Gene ontology analysis revealed significant enrichment in pathways related to the mitotic cell cycle and chromatin organization (Fig. 2E, Table S3). Integration of these transcriptomic changes with predictions from miRanda and RNA22 identified 341 high-confidence piR-775 targets (Fig. S6B–C), including key DNA repair genes such as BARD1, XRCC2, and BRIP1, as well as metastasis-associated regulators like ATOH8, SERPINE1, and CCBE1. Notably, several targets, including TPX2 and BIRC5, are well-established tumor-promoting genes in TNBC [7–9].

CRISPR-Cas9 loss-of-function screening data from DepMap [10] revealed that 47 predicted piR-775 target genes are essential for TNBC cell viability (gene effect score < − 0.3), including POLE, BIRC5, CHAF1B, and others (Fig. 2F). RT-qPCR validation confirmed the downregulation of key targets—TPX2, XRCC2, POLE, and BARD1—in piR-775–overexpressing cells (Fig. 2G). Notably, BARD1 is a critical binding partner of BRCA1 which has been implicated in both familial and sporadic breast cancers through germline mutations and somatic alterations [11, 12].

Comet assays further confirmed that piR-775 overexpression impairs DNA repair in transfected TNBC cells (Fig. S6D). Notably, combined treatment with Olaparib, a PARP inhibitor, and piR-775 mimics led to a dose-dependent increase in cytotoxicity (Fig. 2H), suggesting a potential synthetic lethal interaction. Additionally, expression analysis revealed significant downregulation of PIWIL2 and PIWIL4 in both our patient cohort (Fig. 2I) and in TCGA breast cancer datasets (Fig. S7), indicating broader suppression of the piRNA–PIWI pathway in breast cancer.

In conclusion, piR-775 acts as a tumor-suppressive piRNA that limits TNBC proliferation, migration, invasion, and DNA repair (Fig. 2J), associates with favorable prognosis, and enhances PARP inhibitor efficacy, supporting its potential as a therapeutic target.

## Supplementary Information

Below is the link to the electronic supplementary material.

Supplementary Material 1

Supplementary Material 2

Supplementary Material 3

Supplementary Material 4

Supplementary Material 5

Supplementary Material 6

Supplementary Material 7

Supplementary Material 8

Supplementary Material 9

Supplementary Material 10

Supplementary Material 11

Supplementary Material 12

Supplementary Material 13

## Data Availability

The small RNA transcriptomic data are available in the SRA repository under BioProject number PRJNA953015.

## Abbreviations

TNBC: Triple-negative breast cancer
piRNA: PIWI-interacting RNA
ncRNA: Noncoding RNA
RFS: Relapse-free survival
FC: Fold change
FDR: False discovery rate
RT-qPCR: Reverse transcription quantitative PCR
CFU: Colony forming unit
GFP: Green fluorescent protein
RNA-Seq: RNA sequencing
GO: Gene ontology
3D: Three-dimensional
PIWI: P-element induced wimpy testis
UTR: Untranslated region
TCGA: The Cancer Genome Atlas
CRISPR: Clustered regularly interspaced short palindromic repeats
EtBr: Ethidium bromide
NC: Negative control
