# Supplementary material for: Piwi-interacting RNA 775 (piR-775) predicts favorable prognosis and regulates cell cycle and DNA damage response pathways in breast cancer
Source: Biomark Res. 2025 Nov 4;13:139. doi: 10.1186/s40364-025-00856-1 (PMC12584290; doi:10.1186/s40364-025-00856-1)
Supplement: Supplementary file 1 — Supplementary Material 1 [file 40364_2025_856_MOESM1_ESM.pdf]

Methods:

Study cohort

Detailed description of the study cohort and inclusion and exclusion criteria can be found in our prior publication [1]. Briefly, female patients diagnosed with invasive breast carcinoma who underwent surgical excision of the primary tumor at HMC, Qatar, between 2008 and 2013 were eligible for inclusion. Exclusion criteria included prior neoadjuvant chemotherapy, presence of in-situ malignancy, male gender, recurrent breast cancer, sarcoma, or metastatic carcinoma originating from a non-breast primary site.

Total RNA isolation and NGS

Total RNA isolation from FFPE tissues was described in our previous publication [1]. For small RNA library preparation, 100 ng of total RNA was used as input for 3′ ligation, followed by 5′ ligation and reverse transcription, using the QIAseq miRNA Library Kit (QIAGEN, Hilden, Germany). The resulting libraries were quantified using the Qubit dsDNA HS assay kit and assessed for size distribution by employing the Agilent 2100 Bioanalyzer DNA1000 chip. Pooled libraries underwent sequencing on the Illumina platform. For detection and quantification of piRNA expression (total count), the RNA-Seq data were mapped to piRNA database (https://www.pirnadb.org/download/archive/pirna; Homo sapiens v1.7.6) and the piRNA expression were calculated using the small RNA analysis workflow in CLC Genomics Workbench 24.0.2. Expression data were then imported into iDEP.2.0, normalized (CPM, count per million), and log-transformed using EdgeR (log2(CPM+c)). PiRNAs with a minimum expression of 5 CPM in at least 10 samples were retained. Hierarchical clustering and identification of differentially

expressed piRNAs (DEPs) in relation to PAM50 intrinsic subtypes were conducted in iDEP.2.0 using 1.5 fold change (FC) and false discovery rate (FDR) $p < 0.05$.

Survival analysis

To elucidate the list of piRNAs correlating with ten-year patients' RFS, the normalized piRNA expression data (log2) underwent survival analysis using RStudio 2021.09.2. The 'survival' package in R was employed to compute the log-rank p values and hazard ratios (HR) [2]. Initial identification of potential piRNA candidates associated with RFS was conducted through univariate survival analysis in RStudio and subsequently, these identified piRNA candidates underwent multivariate Cox regression survival analysis using IBM SPSS Statistics v26 to account for potential confounding factors, including molecular subtype, tumor grade, and age. The survival plot was generated by stratifying the patient cohort into high and low groups based on the median piR-775 expression. Log-rank p value was used for curve comparison.

Cell culture and transfection

The human TNBC cell lines (MDA-MB-231 and BT-549) were cultured in Dulbecco's modified Eagle's medium (DMEM) supplemented with D-glucose at a concentration of 4500 mg/L, 2–4 mM L-glutamine, 10% fetal bovine serum, and 1% penicillin-streptomycin (Pen-Strep), all purchased from GIBCO-Invitrogen (Waltham, MA, USA). Cells were cultured as adherent monolayer in a humidified CO_2 (5%) incubator at 37 °C. MCF10A normal breast tissue cell line was maintained in DMEM/F12 media supplemented with mammary epithelial growth supplement (MEGS), 10% fetal bovine serum, and 1% penicillin-streptomycin (Pen-Strep). Cells were transfected with piRNA to conduct several assays. The miRIDIAN mimics of hsa-piR-775, and negative control were purchased from Dharmacon (Lafayette, CO, USA). The reverse

transfection protocol was utilized where piRNA mimics or scrambled control at a final concentration of 30 nM were diluted in 50 µL of Opti-MEM, and 1.5 µL of Lipofectamine 2000 was diluted in 50 µL of Opti-MEM. The resulting mimic and Lipofectamine 2000 mixtures were combined and incubated at room temperature for 20 min. The cell count per well was 1.68×10^5 cells (MDA-MB-231) and 8.4×10^4 cells (BT-549) in 0.6 mL transfection medium (complete DMEM without Pen-Strep). This was followed by the addition of 0.2 mL of the transfection mixture to the 12-well tissue culture plate. After 24 h, the transfection cocktail was topped up with complete DMEM. Transfection of the MCF10A normal breast epithelial cells was carried as detailed above, using DMEM/F-12 media supplemented with FBS, MEGS, Cholera toxin.

Validation of piR-775 overexpression efficiency

To validate piR-775 overexpression efficiency, total RNA was extracted on day three post transfection from both conditions using miRNeasy Mini Kit. The concentration and quality of extracted RNA was measured using NanoDrop 2000 (Thermo Scientific, DE, USA). Following the RNA extraction, reverse transcription PCR was performed using the high-capacity cDNA Reverse Transcription kit and poly(A) tailing kit (applied biosystem). The produced cDNA was used to perform qPCR using the PowerUp SYBR Green Master Mix to measure the level of piRNAs expression in MDA-MB-231. The relative fold change (FC) in piRNA expression was determined using the $2^{-\Delta\Delta Ct}$ method, where the average of ΔCt values for the target amplicon was normalized to that of an endogenous control (hsa-miR-21-5p) and compared with negative control transfected samples. Custom piRNA primers were designed using http://www.srnaprimerdb.com/. The list of primers used in current study are listed in Table S1.

Colony Formation Unit (CFU) Assay

For piRNA transfection, plates were monitored until the negative control condition became nearly confluent. The CFU assay was employed to assess the ramifications of exogenous expression of piRNA candidates on TNBC Cells. Cells were washed two times with phosphate-buffered saline (PBS). Subsequent staining with crystal violet (0.5% in 20% methanol) was done and plates were placed on the shaker for 2-3 hours. Plates were then air-dried at room temperature before imaging, and CFUs were quantified by dissolving crystal violet in 10% SDS, and subsequent measurement of absorbance at 340λ.

GFP-labelling of tumor cells and Time course cell proliferation assay

To track tumor cells in cell-based assays, tumor cell line (MDA-MB-231) was engineered to stably expressed GFP by transduction of a pre-made eGFP-puro-CMV-lentiviral vector (GeneCopoeia) as we described before [3]. GFP-labelled MDA-MB-231 cells were reverse transfected with selected piRNA candidates and non-targeting control piRNAs at final concentration of 24 nM and then were seeded at a density of 7,000 cells in 96-well format plates. The next day, the media was replaced with fresh growth media. Plates were imaged immediately (T0) and at 24 h intervals over a period of 96 h using a controlled environment chamber of the CLS Operetta high content imaging system (PerkinElmer). Nine view fields were acquired for each well using a 10x objective. GFP labelled cell number was quantified using Harmony® image analysis software. To calculate the cell growth rate, cell count at each time point was normalized to its respective replicate at T0.

Tumor cell 2D migration assay

GFP-labelled MDA-MB-231 cells were first transfected with piR-775 mimic and non-targeting RNA control at final concentration of 24 nM. Next, the transfected cells were cultured at

25,000 cells per well in 96-well plates fitted with stoppers (Oris™ Cell Migration Assay, Platypus Technologies). Cells were incubated overnight at 37°C and 5% CO_2 before removing the stoppers. The plates were imaged immediately (T0) and 120 h upon removal of stoppers using the CLS Operetta high content imaging system (PerkinElmer). Nine view fields were acquired for each well using a 10x objective. The view field image tiles were stitched into one image for quantification. The number of migrated cells into the gap was quantified using Harmony® image analysis software and NIH Image J software as we described before [3].

Tumor cell invasion on-a chip assay

To assess the effect of piRNA candidates on cancer cell invasion, we used the OrganoPlate®-3-lane (Mimetas), a microfluidic organ-on-chip platform as described previously [3] (figure 4. A). Briefly, 2.2 µL of type I collagen with a final concentration of 5 mg/mL (stock solution of 10 mg/mL rat tail type I collagen (Corning) was neutralized with 10% 37 g/l Na_2CO_3 (pH 9.5) and 10% 1 M HEPES buffer) and was loaded in the middle channel of the chip. The plate was incubated for 15 min at 37°C, 5% CO2, until the polymerization of collagen gel. Next, 40 µL of fibronectin in PBS with a final concentration of 10 µg/mL was added into one of the perfusion channels. The next day, GFP-labeled MDA-MB-231 cells were resuspended in DMEM media at concentration of $8x10^6$ cells/mL. 4 µL of cell suspension was seeded into the inlet of the perfusion channel. OrganoPlates were then incubated for 2-3 hours at 37°C, 5% CO_2 until complete cell attachment. Then, 50 µL of DMEM growth media was added into the inlet and outlet of tumor perfusion channel. 50 µL of EGM-2 growth media to the opposite perfusion channel. Plates were moved to the Mimetas rocker platform in the cell culture incubator to generate a bi-directional flow by leveling at a 14° angle and 8 min interval. These settings were maintained during the experiment period. The following day, cells were transfected with the selected piRNA candidates

and the non-targeting piRNA controls (NC) by perfusing the transfection complex: piRNA/transfection reagent at final concentration of 24 nM. The media was then changed after 24 hours and refreshed later every 48 hours. After 5 days of piRNA transfection, OrganoPlates® were fixed using 4% paraformaldehyde in PBS for 15 min and washed three times with PBS for 5 min. OrganoPlates® were incubated with permeabilization buffer containing Triton 0.05% in PBS for 30 min. Next, the chips were incubated with Hoechst 33342 (Thermo Fisher Scientific) for nuclear staining, Alexa-568 Phalloidin (Thermo Fisher Scientific) for actin fiber staining and Deep Red Cell Mask (Thermo Fisher Scientific) to delineate the cell body. OrganoPlates® were then imaged with a 10× objective using the CLS operetta high content microscopy system (PerkinElmer). Images of chip channels were acquired using spinning disk confocal mode with 50 z-steps with 5 µm spacing. Four adjacent view fields were acquired to cover both chip channels. The images were stacked and stitched to perform the image segmentation and quantification using the built-in Harmony® image analysis software. The invaded tumor cell number was normalized to the total tumor cells seeded in the perfusion channel for each replicate.

Identification of potential gene targets for piR-775

To identify potential gene targets for piR-775, MDA-MB-23 cells were transfected with piRNA mimic or negative control as described above. On the third day post-transfection, total RNA was extracted, and subsequently library preparation was done using the TruSeq Stranded Total RNA Library (Illumina Inc., San Diego, CA, USA) as we described before [4]. The generated FASTQ files were subsequently aligned and mapped to the hg38 reference genome using the CLC Genomics Workbench 20.2. Subsequently, iDEP.951 was employed for differential expression analysis to identify differentially expressed genes in piR-775 overexpressing TNBC model. The 3' UTR of downregulated genes were subsequently retrieved from Ensembl database and were

subsequently aligned to piR-775 sequence using Miranda 3.3a [5]. Predicted gene targets were then used for STRING PPI enrichment analysis [6].

Exploration of the essentiality of piR-775 targets in TNBC

The gene effect scores for identified piR-775 targets were retrieved from the genome-wide CRISPR-Cas9 functional screen data from the Achilles project in TNBC models as described before [7].

RT-qPCR for piR-775 gene targets

The candidate genes identified as bona fide targets for hsa-piR-775 were validated in MDA-MB-231 post piRNA mimic transfection using qRT-PCR. RNA extracted from MDA-MB-231 (500 ng) were reverse transcribed to cDNA using the High-Capacity cDNA Reverse Transcription kit (Thermo Fisher Scientific, Waltham, MA). Subsequently, qPCR was performed with specific primer pairs as detailed in Table S1 and the PowerUp SYBR Green Master Mix (Thermo Fisher Scientific, Waltham, MA) on QuantStudio 7 Flex qPCR system (Applied Biosystems). The mRNA transcript levels of the target genes were determined based on their respective CT values, normalized against β-actin (ACTB) transcript levels, and presented as fold change using the delta delta CT method compared to control cells.

Comet Assay

Cells, transfected with piR-775 or control mimic, were treated with 10 mM H_2O_2 for 5 minutes, rinsed once with complete medium, and allowed to recover at 37 °C for 0 and 4 hours. Following recovery, cells were harvested using trypsin for 4 minutes, and trypsin activity was neutralized with complete growth medium. The cells were collected in 1.5 mL tubes and

centrifuged at 300 × g for 5 minutes. After the aspiration of the supernatant, the cell pellet was resuspended in cold PBS at a concentration of 10^5 cells/mL.

An aliquot of 30 µL from the cell suspension was mixed with 270 µL of 1% low-melting-point agarose in PBS at 37 °C. Then, 50 µL of this mixture was layered onto a microscope slide pre-coated with a thin layer of 1% low-melting-point agarose. The slides were kept at 4 °C for 30 minutes before immersion in cold lysis solution (2.5 M NaCl, 100 mM EDTA, 10 mM Tris, 1% sodium lauryl sarcosinate, 1% Triton X-100, and 200 mM NaOH, pH 10). After 1 hour in the dark at 4 °C, slides were transferred to alkaline electrophoresis buffer (1.2% NaOH, 1 mM EDTA) for DNA unwinding (1 hour), followed by electrophoresis at 25 V/300 mA for 40 minutes. Upon completion of electrophoresis, the slides were neutralized in 0.4 M Tris buffer (pH 7.5) for three 5-minute washes, then immersed in 70% ethanol for 30 minutes and air-dried at 37 °C for 15 minutes. Finally, the slides were stained with SYBR Green and analyzed using a Zeiss fluorescence microscope.

Combination Treatment with Olaparib

Olaparib (Selleck Chemicals, Houston, TX, USA) was reconstituted in DMSO at a stock concentration of 10 mM and used at final concentrations of 5 µM and 10 µM. To assess the combined effect of Olaparib and piR-775, cells were seeded in 12-well plates at a density of 0.168×10^6 cells per well and transfected with piR-775 at a final concentration of 15 nM, as previously described. Cells were then treated with Olaparib in complete growth medium under the indicated conditions. Colony formation assays were performed on day 6 following drug treatment.

References

1. Elango R, Rashid S, Vishnubalaji R, Al-Sarraf R, Akhtar M, Ouararhni K, Decock J, Albagha OM, Alajez NM: **Transcriptome profiling and network enrichment analyses identify subtype-specific therapeutic gene targets for breast cancer and their microRNA regulatory networks**. *Cell Death & Disease* 2023, **14**(7):415.
2. Therneau TM, Grambsch PM, Therneau TM, Grambsch PM: **The cox model**: Springer; 2000.
3. Ozer LY, Fayed HS, Ericsson J, Al Haj Zen A: **Development of a cancer metastasis-on-chip assay for high throughput drug screening**. *Frontiers in Oncology* 2024, **13**:1269376.
4. Vishnubalaji R, Awata D, Alajez NM: **LURAP1L-AS1 Long Noncoding RNA Promotes Breast Cancer Progression and Associates with Poor Prognosis**. *Non-coding RNA Research* 2025.
5. Enright A, John B, Gaul U, Tuschl T, Sander C, Marks D: **MicroRNA targets in Drosophila**. *Genome biology* 2003, **4**:1-27.
6. Szklarczyk D, Gable AL, Nastou KC, Lyon D, Kirsch R, Pyysalo S, Doncheva NT, Legeay M, Fang T, Bork P *et al*: **The STRING database in 2021: customizable protein-protein networks, and functional characterization of user-uploaded gene/measurement sets**. *Nucleic Acids Res* 2021, **49**(D1):D605-D612.
7. Meyers RM, Bryan JG, McFarland JM, Weir BA, Sizemore AE, Xu H, Dharia NV, Montgomery PG, Cowley GS, Pantel S *et al*: **Computational correction of copy number effect improves specificity of CRISPR-Cas9 essentiality screens in cancer cells**. *Nat Genet* 2017, **49**(12):1779-1784.
