# Supplementary material for: Piwi-interacting RNA 775 (piR-775) predicts favorable prognosis and regulates cell cycle and DNA damage response pathways in breast cancer
Source: Biomark Res. 2025 Nov 4;13:139. doi: 10.1186/s40364-025-00856-1 (PMC12584290; doi:10.1186/s40364-025-00856-1)
Supplement: Supplementary file 2 — Supplementary Material 2 [file 40364_2025_856_MOESM2_ESM.pdf]

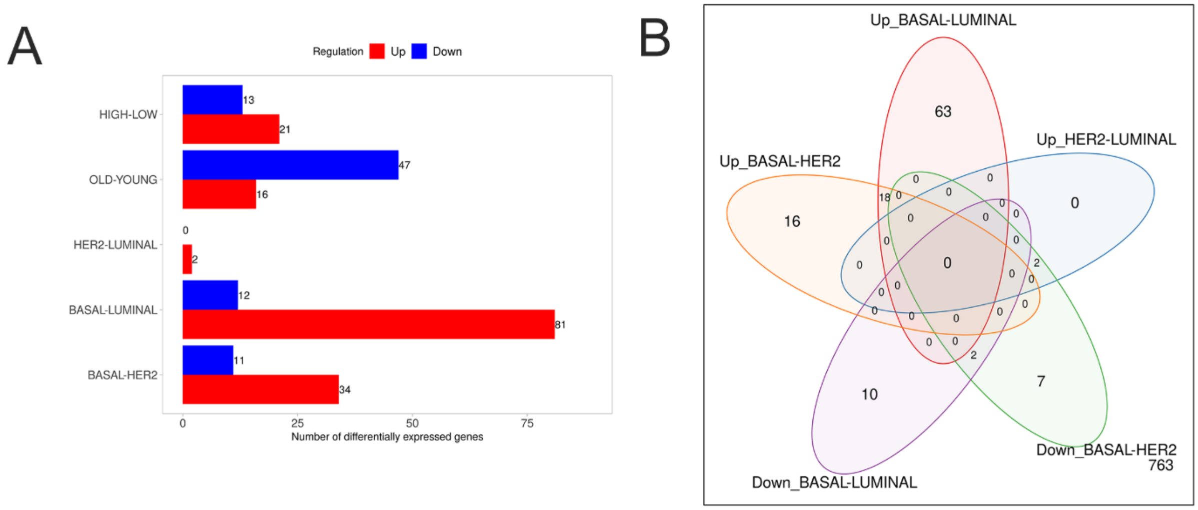

Figure S1. Differential expression of piRNAs across breast cancer subtypes. A) Bar graph showing the number of differentially expressed piRNAs (FDR < 0.05, fold change ≥ 1.5) identified across various comparisons, after adjusting for confounding variables**. B)** Venn diagram illustrating the overlap of upregulated and downregulated piRNAs among different intrinsic breast cancer subtypes.
