# Supplementary material for: Piwi-interacting RNA 775 (piR-775) predicts favorable prognosis and regulates cell cycle and DNA damage response pathways in breast cancer
Source: Biomark Res. 2025 Nov 4;13:139. doi: 10.1186/s40364-025-00856-1 (PMC12584290; doi:10.1186/s40364-025-00856-1)
Supplement: Supplementary file 3 — Supplementary Material 3 [file 40364_2025_856_MOESM3_ESM.pdf]

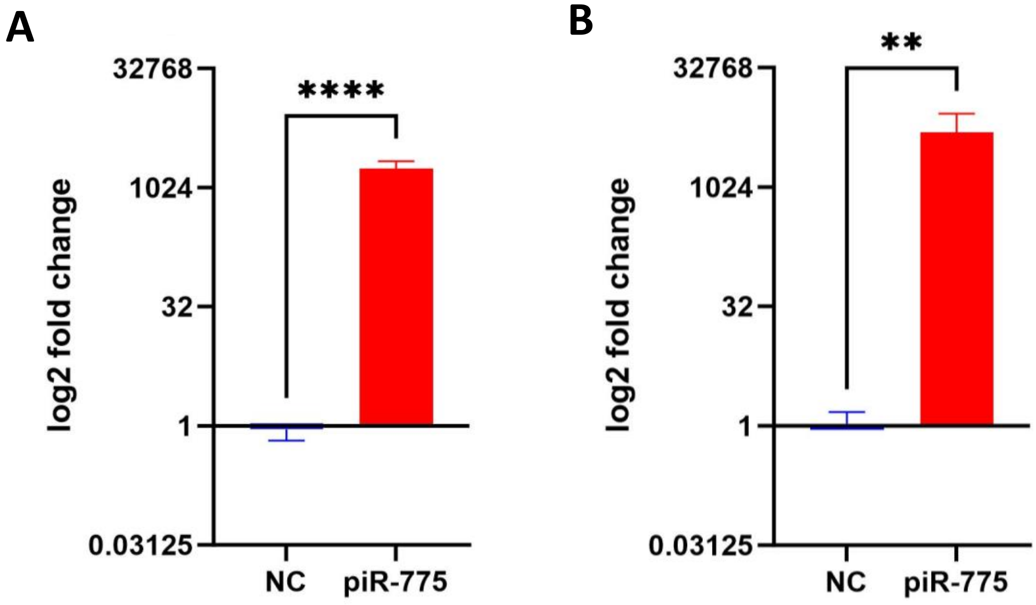

Figure S2. Expression of piR-775 in transfected TNBC cells. RT-qPCR quantification of piR-775 expression in MDA-MB-231 **(A)** and BT-549 **(B)** TNBC cells transfected by piR-775 mimic

compared to control cells. Data are presented as mean ± S.D.
