# Supplementary material for: Piwi-interacting RNA 775 (piR-775) predicts favorable prognosis and regulates cell cycle and DNA damage response pathways in breast cancer
Source: Biomark Res. 2025 Nov 4;13:139. doi: 10.1186/s40364-025-00856-1 (PMC12584290; doi:10.1186/s40364-025-00856-1)
Supplement: Supplementary file 4 — Supplementary Material 4 [file 40364_2025_856_MOESM4_ESM.pdf]

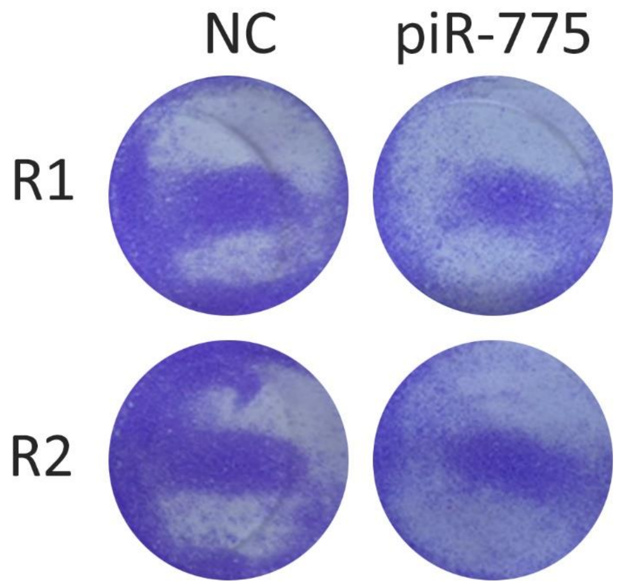

Figure S3. CFU of MCF10A transfected with control (NC) or piR-775 mimic.
Two representative images are displayed for each condition.
