# Supplementary material for: Piwi-interacting RNA 775 (piR-775) predicts favorable prognosis and regulates cell cycle and DNA damage response pathways in breast cancer
Source: Biomark Res. 2025 Nov 4;13:139. doi: 10.1186/s40364-025-00856-1 (PMC12584290; doi:10.1186/s40364-025-00856-1)
Supplement: Supplementary file 5 — Supplementary Material 5 [file 40364_2025_856_MOESM5_ESM.pdf]

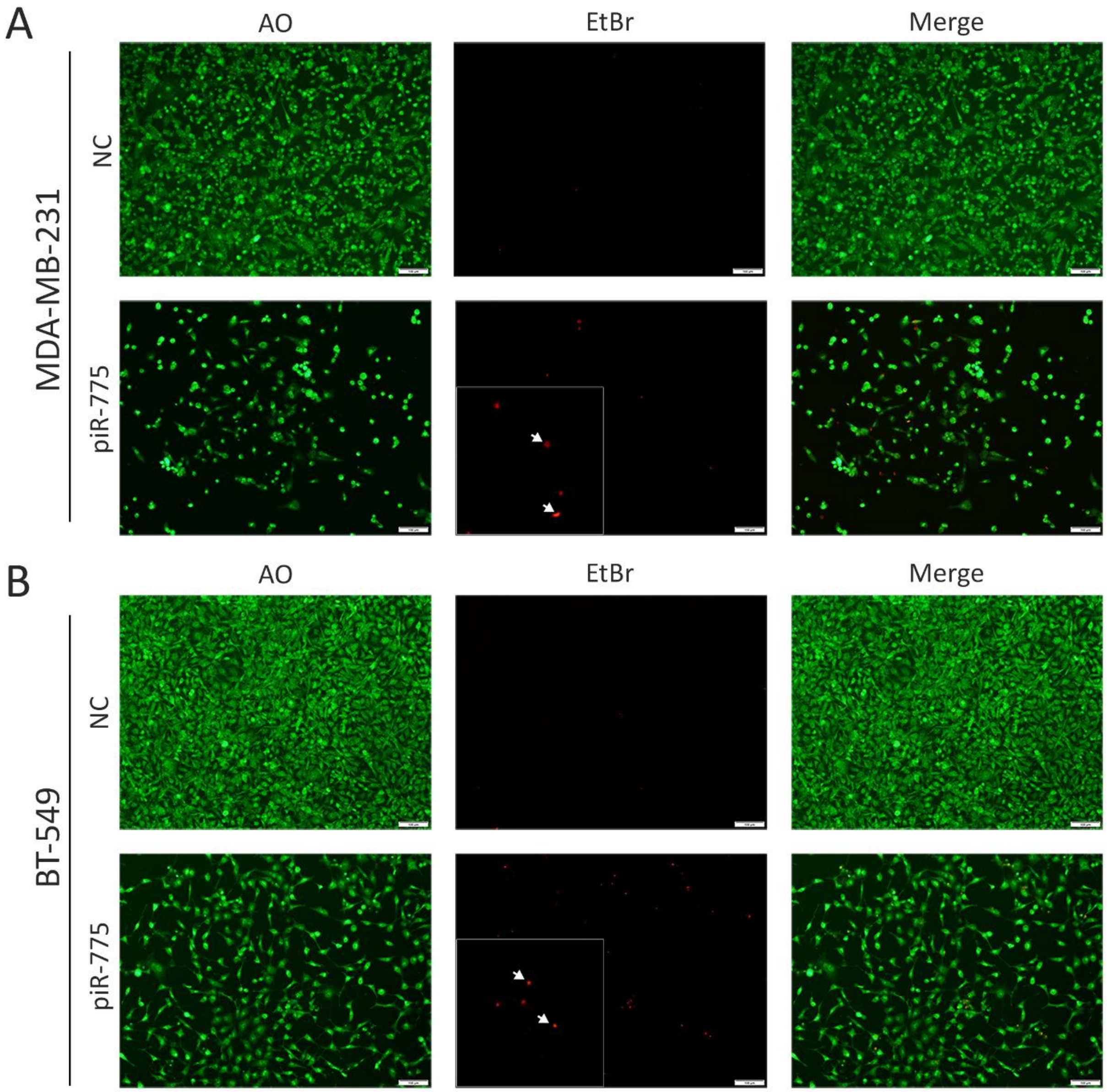

Figure S4. Dead-live staining of TNBC cells in response to exogeneous expression of piR-775 mimics. Representative fluorescence images for MDA-MB-231 **(A)** and BT-549 **(B)** TNBC models post transfection with piR-775 mimics compared to negative control. Cells were stained on day 5 with AO/ EtBr to detect dead cells (red; necrotic).
