# Supplementary material for: Piwi-interacting RNA 775 (piR-775) predicts favorable prognosis and regulates cell cycle and DNA damage response pathways in breast cancer
Source: Biomark Res. 2025 Nov 4;13:139. doi: 10.1186/s40364-025-00856-1 (PMC12584290; doi:10.1186/s40364-025-00856-1)
Supplement: Supplementary file 6 — Supplementary Material 6 [file 40364_2025_856_MOESM6_ESM.pdf]

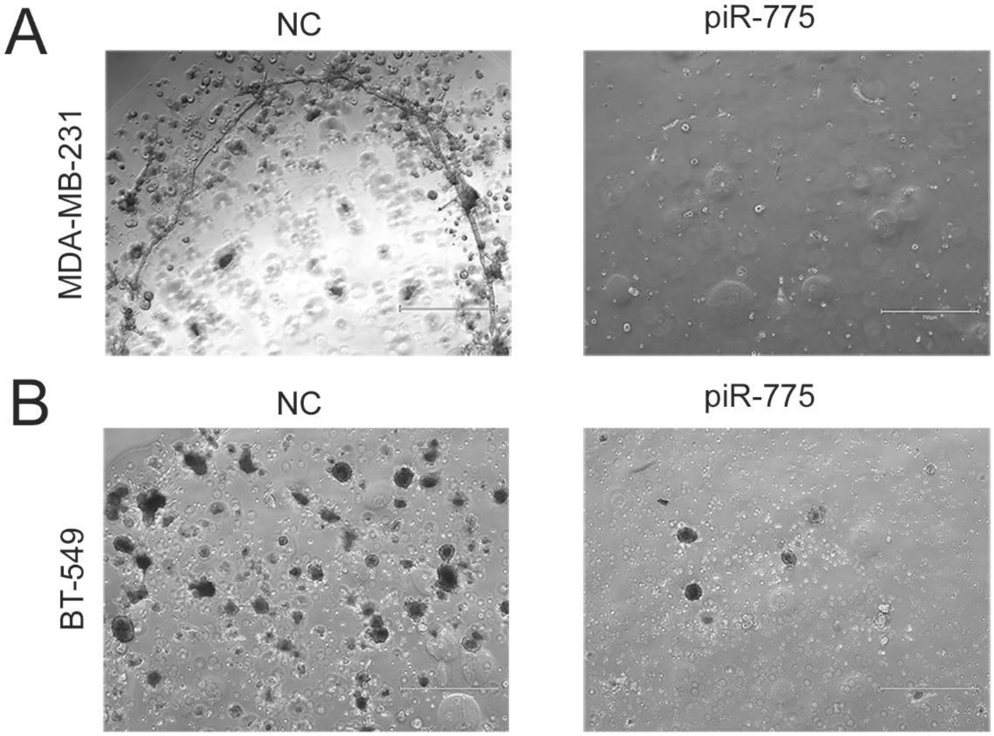

Figure S5. piR-775 suppresses TNBC cell growth in 3D culture models. Representative images showing reduced tumor cell growth in 3D Matrigel culture following piR-775 mimic transfection in MDA-MB-231 **(A)** and BT-549 **(B)** triple-negative breast cancer cells.
