# Supplementary material for: Piwi-interacting RNA 775 (piR-775) predicts favorable prognosis and regulates cell cycle and DNA damage response pathways in breast cancer
Source: Biomark Res. 2025 Nov 4;13:139. doi: 10.1186/s40364-025-00856-1 (PMC12584290; doi:10.1186/s40364-025-00856-1)
Supplement: Supplementary file 7 — Supplementary Material 7 [file 40364_2025_856_MOESM7_ESM.pdf]

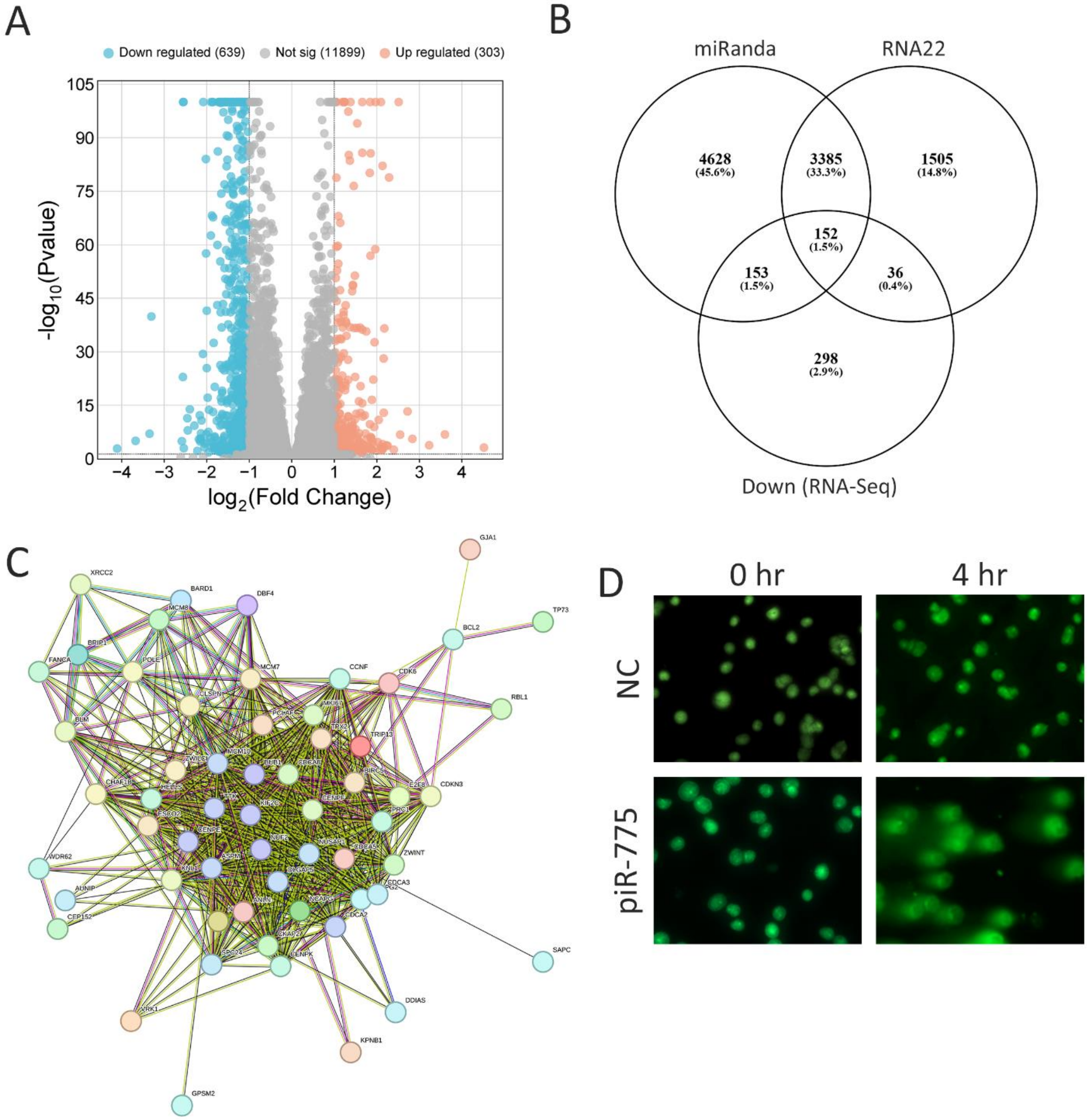

Figure S6. Transcriptomic and functional identification of piR-775 target genes. A) Volcano plot displaying differentially expressed genes in MDA-MB-231 cells transfected with piR-775 mimics versus negative control. **B)** Venn diagram showing the overlap between computationally predicted piR-775 targets (identified using miRanda and RNA22) and significantly downregulated genes upon piR-775 overexpression. **C)** STRING network illustrating cell cycle–related genes among the predicted piR-775 targets. **D)** Representative images from comet assay demonstrating delayed DNA damage repair in TNBC cells overexpressing piR-775 compared to controls.
