# Supplementary material for: Piwi-interacting RNA 775 (piR-775) predicts favorable prognosis and regulates cell cycle and DNA damage response pathways in breast cancer
Source: Biomark Res. 2025 Nov 4;13:139. doi: 10.1186/s40364-025-00856-1 (PMC12584290; doi:10.1186/s40364-025-00856-1)
Supplement: Supplementary file 8 — Supplementary Material 8 [file 40364_2025_856_MOESM8_ESM.pdf]

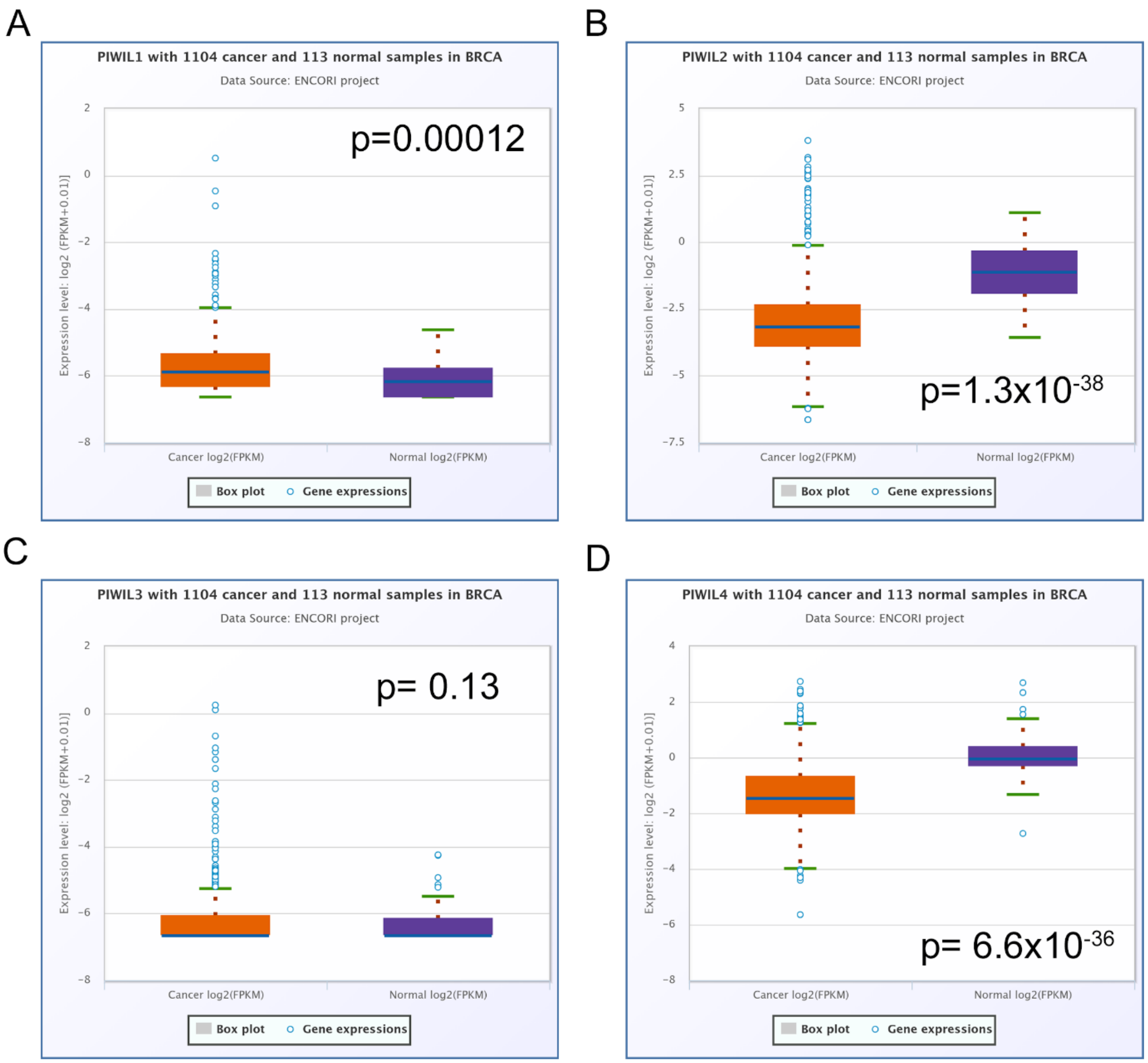

Figure S7. Differential expression of PIWI family members in breast cancer versus normal breast tissue based on the ENCORI (StarBase) dataset. Box plots show the expression levels (log2[FPKM+0.01]) of **PIWIL1 (A)**, **PIWIL2 (B)**, **PIWIL3 (C)**, and **PIWIL4 (D)** across breast cancer (BRCA; $n = 1104$) and normal breast tissue samples ($n = 113$). Statistical comparisons between groups were performed using a two-tailed *t*-test. **PIWIL2** and **PIWIL4** were significantly downregulated in breast cancer, with highly significant *p*-values ($p = 1.3 \times 10^{-38}$ and $p = 6.6 \times 10^{-36}$, respectively), while **PIWIL1** showed moderate downregulation ($p = 0.00012$), and **PIWIL3** expression differences were not statistically significant ($p = 0.13$). These findings further support the reduction of PIWI protein expression in breast cancer.
