# Supplementary material for: Piwi-interacting RNA 775 (piR-775) predicts favorable prognosis and regulates cell cycle and DNA damage response pathways in breast cancer
Source: Biomark Res. 2025 Nov 4;13:139. doi: 10.1186/s40364-025-00856-1 (PMC12584290; doi:10.1186/s40364-025-00856-1)
Supplement: Supplementary file 13 — Supplementary Material 13 [file 40364_2025_856_MOESM13_ESM.docx]

| Table S1. Primers used in current study for RT-PCR and qPCR | | |
| --- | --- | --- |
| S. No. | Name | Sequence |
| 1 | BARD1 | F: CTGCTCGCGTTGTACTAACAT  R: TCCAATGCAGTCACTTACACAAT |
| 2 | TPX2 | F: GGAAGCACCAGCTGGAAGAA  R: AGAGAACCAGAAAGGCCCTC |
| 3 | POLE | F: TTCCTCAGTTTCGGCACTCAA  R: CTCAAAACCAAACCGCAAATCC |
| 4 | XRCC2 | F: TGCTTTATCACCTAACAGCACG  R: TGCTCAAGAATTGTAACTAGCCG |
| 5 | BIRC5 | F: CAGACTTGGCCCAGTGTTTC  R: GTTCCTCTATGGGGTCGTCA |
| 6 | β-actin | F: GGCACCCAGCACAATGAAG  R: CCGATCCACACGGAGTACTTG |
| 7 | hsa-piR-775 | AACAAGACTGTGTGCTGATTGTCAC |
| 8 | hsa-miR-21-5p | AAGCGACCTAGCTTATCAGACT |
| 9 | Universal RT primer | CAGTGCAGGGTCCGAGGTCAGAGCCACCTGGGCAATTTTTTTTTTTVN |
| 10 | Rp | CAGTGCAGGGTCCGAGGT |
